# Influence of 1α, 25-dihydroxyvitamin D_3_ [1, 25(OH)_2_D_3_] on the expression of Sox 9 and the transient receptor potential vanilloid 5/6 ion channels in equine articular chondrocytes

**DOI:** 10.1186/s40781-014-0033-1

**Published:** 2014-12-17

**Authors:** Ismail M Hdud, Paul T Loughna

**Affiliations:** School of Veterinary Medicine and Science, Faculty of Medicine and Health Sciences, University of Nottingham, Sutton Bonington Campus, Leicestershire, LE12 5RD UK; Medical Research Council-Arthritis Research UK Centre for Musculoskeletal Ageing Research, University of Nottingham, Leicestershire, UK; School of Veterinary Medicine and Science, Tripoli University, Tripoli, Libya

**Keywords:** Cartilage, Vitamin D, Sox 9, TRPV, Chondrocyte

## Abstract

**Background:**

Sox 9 is a major marker of chondrocyte differentiation. When chondrocytes are cultured *in vitro* they progressively de-differentiate and this is associated with a decline in Sox 9 expression. The active form of vitamin D, 1, 25 (OH)_2_D_3_ has been shown to be protective of cartilage in both humans and animals. In this study equine articular chondrocytes were grown in culture and the effects of 1, 25 (OH)_2_D_3_ upon Sox 9 expression examined. The expression of the transient receptor potential vanilloid (TRPV) ion channels 5 and 6 in equine chondrocytes *in vitro*, we have previously shown, is inversely correlated with de-differentiation. The expression of these channels in response to 1, 25 (OH)_2_D_3_ administration was therefore also examined.

**Results:**

The active form of vitamin D (1, 25 (OH)_2_D_3_) when administered to cultured equine chondrocytes at two different concentrations significantly increased the expression of Sox 9 at both. In contrast 1, 25 (OH)_2_D_3_ had no significant effect upon the expression of either TRPV 5 or 6 at either the protein or the mRNA level.

**Conclusions:**

The increased expression of Sox 9, in equine articular chondrocytes *in vitro*, in response to the active form of vitamin D suggests that this compound could be utilized to inhibit the progressive de-differentiation that is normally observed in these cells. It is also supportive of previous studies indicating that 1α, 25-dihydroxyvitamin D_3_ can have a protective effect upon cartilage in animals *in vivo*. The previously observed correlation between the degree of differentiation and the expression levels of TRPV 5/6 had suggested that these ion channels may have a direct involvement in, or be modulated by, the differentiation process *in vitro*. The data in the present study do not support this.

## Background

The active metabolite of vitamin D, 1, 25 (OH)_2_D_3_ has been reported to be involved in the regulation of cellular functions such as differentiation, proliferation, and the immune system [1]. Similar to other steroids, 1, 25 (OH)_2_D_3_ exerts physiological actions at two levels by genomic and non-genomic mechanisms. The genomic mechanism exerts its effect on the cells by binding to specific receptors known as vitamin D receptors, which in turn bind to retinoid X receptors to form heterodimeric complexes [2]. These heterodimeric complexes in turn bind to VDREs (vitamin D responsive elements) of the upstream region of the responsive gene leading to activation/suppression of the target gene. In contrast, the non-genomic effect of 1, 25 (OH)_2_D_3_ is mediated though plasma membrane receptors of the target cells which are involved in ion channel activity regulation and also activate signal transduction pathways [3,4]. Exogenous 1, 25 (OH)_2_D_3_ in mice increased chondrocyte proliferation as well as enhancing cartilage matrix mineralization [5]. The profound effects of vitamin D on calcium metabolism are well established and it has been reported that it can protect against cartilage loss in osteoarthritis in humans and rats [6].

Sox 9 (Sry-type high mobility group domain) transcription factor is expressed in articular chondrocytes, central nervous and urogenital systems [7,8]. It is expressed in all primordial cartilage tissues during embryogenesis [8,9] and functions to induce differentiation of stem cells to chondrocytes [10], regulate cartilage development and phenotypic maintenance during embryonic development [11]. Studies indicate that Sox 9 is involved in chondrocyte differentiation by regulating the expression of cartilage-specific genes such as collagen type IIα1 [8,12], XIα2 [13], aggrecan [14], collagen link protein [15], cartilage oligomeric matrix protein and Cd-rap [16]. It has been shown that Sox 9 expression is maintained in articular chondrocytes and reduced in osteoarthritic chondrocytes [17].

Haploinsufficiency of Sox 9 results in campomelic dysplasia, a severe syndrome resulting in inadequate cartilage formation during development leading to severe dwarfism [18]. In chimaeric mice, Sox 9^−/−^ the prechondrogenic mesenchyme cells were prevented from differentiating into chondrocytes and lost their ability to express chondrocyte specific genes [19,20]. Sox 9 expression is also significantly reduced in osteoarthritic cartilage compared to normal healthy cartilage [17,21].

*In vitro* propagation of articular chondrocytes results in de-differentiation that is characterized by gradual loss of chondrocytic phenotype and acquisition of a fibroblastic phenotype [22] which is associated with a rapid decline in Sox 9 expression [23]. We have previously shown in equine chondrocytes that there is an association between the degree of de-differentiation and the expression and the transient receptor potential vanilloid (TRPV) channels 5 and 6 [24].

The transient receptor potential (TRP) superfamily is a non-selective cation ion channels with relative calcium selectivity. The TRPV sub-family is divided into two groups. TRPV1-4 channels are non-selective ion channels with modest permeation to calcium. This group can be activated by different stimuli such as heat/cold, chemical/mechanical stresses and binding to second messengers [25,26]. The other group comprises of TRPV5 and TRPV6 channels that are highly calcium selective and tightly regulated by cytosolic Ca^2+^ concentration [27,28]. The TRPV5 channel is implicated in Ca^2+^ reabsorption from the kidney, whereas TRPV6 channel is involved in Ca^2+^ absorption in the intestine [29]. Expression of both channels in human articular chondrocytes at mRNA level has been reported [30]. We also demonstrated their expression at the protein levels in equine articular chondrocytes (EAC) [24]. A correlation between the expression of TRPV5/6 channels and administered 1, 25 (OH)_2_D_3_ concentration has also reported in intestinal endothelial cells [31,32], renal cells [33], osteoblasts [34,35]. Calbindin-D9K is a cytosolic Ca^2+^ binding protein, a member of cellular proteins found in the cells with high affinity for Ca^2+^ ions. Calbindin-D9k knockout mice models demonstrated that 1, 25 (OH)_2_D_3_ intake increased the expression of both channels [36]. The aim of the present study was to examine the effects of 1, 25 (OH)_2_D_3_ on the expression of Sox 9 and TRPV 5 and 6 in cultured equine chondrocytes.

## Methods

### Chondrocyte isolation

Articular chondrocytes were isolated from equine articular cartilage removed from load bearing synovial joints (metacarpophalangeal joints) of skeletally mature animals obtained on the day of slaughter from a local abattoir; these animals were euthanized for purposes other than research. All experiments were performed with local institutional ethical approval (University of Nottingham, School of Veterinary Medicine and Science Ethical Committee), in strict accordance with national guidelines. Articular cartilage slices were collected in serum free DMEM medium supplemented with 2% antibiotics (50 U/ml penicillin and 50 μg/ml streptomycin) (Invitrogen, UK). Cartilage slices were washed in phosphate buffer saline (PBS) supplemented with 10% antibiotics (50 U/ml penicillin and 50 μg/ml) for 30 min with agitation, followed by enzymatic digestion in freshly prepared 0.1% (v/w) collagenase type I from *clostridium histolyticum* enzyme dissolved in serum free medium supplemented with 2% antibiotics at relative humidity of 95%, 5% CO_2_, 37°C for 18 h. Undigested cartilage debris were removed from the cell/medium suspension by filtering the mixture through a nylon filter strainer of 70 μm pore size (BD Bioscience, Europe). Supernatant was spun to isolate the chondrocytes, followed by three washes using PBS containing 10% antibiotics. Finally cells were suspended in DMEM contains 2% antibiotics and 10% FCS and cultured at 37°C, 95% humidity and 5% CO_2_ until confluent. Cells viability was assessed by the trypan blue exclusion test (Sigma Aldrich, UK).

### Vitamin D treatment

To explore the influence of the active form of vitamin D (1, 25, α-dihydroxy vit D) on equine articular chondrocytes, cells were cultivated at 2 × 10^5^ cells/well until sub-confluence. Chondrocytes were treated with different concentrations of 1, 25 (OH)_2_D_3_ (1 × 10^−9^ and 1 × 10^−12^) [37] in serum free-medium for 24 h. The concentration of DMSO was below 0.1%. At the end of 1, 25 (OH)_2_D_3_ treatment, cells were washed three times sterile PBS and whole cell lysate was collected for protein expression using western blotting.

### Western blotting

Total cellular protein lysate was isolated using radio-immunopreceptation assay (RIPA) buffer (150 mM NaCl, 50 mM Tris–HCl, pH 7.5, 5 mM ethylene glycol tetraacetic acid (EGTA), 1% Triton, 0.5% sodium deoxycholate and 0.1% sodium dodecyl sulphate) supplemented with phosphatase and protease inhibitors cocktail (Roche Diagnostic, Mannheim, Germany) on ice. Protein concentration was quantified by the Bradford method. 25 μg of total protein lysate was mixed with sample buffer (0.5 M Tris–HCl, pH 6.8, 100% glycerol, 20% SDS, 0.5% bromophenol blue and 5% β-mercaptoethanol), separated on a 4/1-% polyacrylamide gel, then electrically transferred to PVDF membrane (Invitrogen, UK) by semi-dry apparatus (Bio-Rad, UK). Transferred proteins were blocked for non-specific binding in 5% (w/v) non-fat milk diluted in Tris base buffer saline with 0.1% tween 20 (TBS-T), followed by incubation with designated antibodies overnight. Membranes were washed five times in TBST, followed by 1 h incubation with goat anti-rabbit IgG conjugated with horseradish peroxidase (HRP) (Dako, UK) secondary antibody at room temperature. Finally, five washes were carried out for 5 min each followed by developing the membrane with the Amersham ECL western blot enhanced chemiluminescence kit (GE Healthcare, UK) and visualized by exposing to X-ray film.

### Quantitative PCR

The quantitative real time PCR was carried out using a LightCycler® 480 PCR System (Roche Diagnostics) using SYBR green DNA-binding fluorescent dye. 20 μl reactions were made in an optical 96-well reaction plate in triplicate and contained: a mixture of template cDNA (5 μl), sense and anti-sense gene specific primers (0.8 μl) (20 pmol), SYBR Green detection reagent and 3.4 μl RNA-free water. Reaction plate was sealed with ABI-prism optical adhesive cover, and spun at 2000 rpm for 2 min. The expression of target genes were normalised against GAPDH (Glyceraldehyde 3-phosphate dehydrogenase) and HPRT (Hypoxanthine-guanine phosphoribosyltransferase) using comparative cycle of threshold (Ct) value method.

### Statistical analysis

Data values are presented as the mean ± SEM. Each experiment was performed in triplicate. The relative expression on the graphs represents the mean of a combination of three experiments. The differences between animals were analyzed utilizing Student’s *t*-test followed by the Bonferroni correction. *P* values less than or equal to 0.05 were considered statically significant.

## Results and discussion

The active form of vitamin D (1, 25 (OH)_2_D_3_) has been shown to play an important role in Ca^2+^ homeostasis. Intracellular Ca^2+^ concentration is involved in several chondrocyte functions including ECM biosynthesis. This study was designed to explore the possible influence of the active form of vitamin D (1, 25 (OH)_2_D_3_) treatment on the expression of Sox 9 and members of the TRP subfamily that are known as epithelial Ca^2+^ channels (TRPV5 and TRPV6 channels).

**Figure 1:**
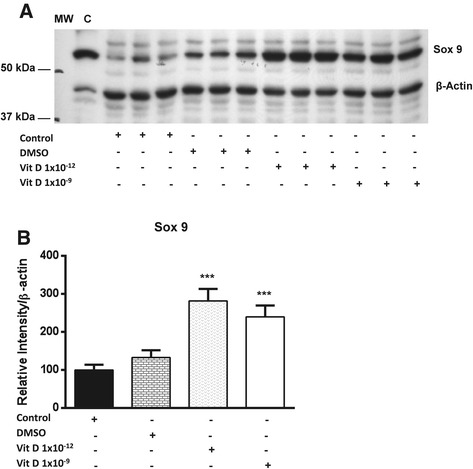
**The effect of 1, 25 (OH)**_**2**_ **D**_**3**_ **on protein expression of Sox 9 in equine articular chondrocytes following 24 h of incubation. (A)** Western blot analysis of protein expression levels using an antibody raised against the Sox 9 protein. Freshly isolated equine whole chondrocyte lysate was used as a positive control C. β-actin was used as an internal load control. **(B)** the level of expression of the protein following 24 h of incubation with different doses (1 × 10^−9^ and 1 × 10^−12^) of 1, 25 (OH)_2_D_3_ treatment in addition to the solvent (DMSO). Values are presented as means ± S.E. ***Indicates statistically significant at *P* < 0.001.

The influence of two doses (1 × 10^−9^ and 1 × 10^−12^) of 1, 25 (OH)_2_D_3_ treatment for 24 h on the expression of the transcription factor (Sox 9) at the protein level in equine chondrocytes was examined. Incubation of chondrocytes for 24 h with 1 × 10^−12^ of 1, 25 (OH)_2_D_3_ significantly increased the expression of Sox 9 by nearly 3 fold, whereas treatment with 1 × 10^−9^ of 1, 25 (OH)_2_D_3_ induced more than 2 fold increase (P < 0.001) compared to the non-treated cells (Figure 1).

In chondrocytes, the mechanisms by which Sox 9 expression is regulated is of great interest due to the critical role played by this transcription factor in controlling the chondrocyte phenotype. The findings of the present study shows an elevation of Sox 9 protein in response to treatment with 1, 25 (OH)_2_D_3_. Isolation of chondrocytes results in their de-differentiation and loss of chondrocyte phenotype to fibroblast phenotype [22]. Recent studies have reported changes in the pattern of expression of some proteins during the course of chondrocytes de-differentiation. Cultivation of de-differentiated chondrocytes in alginate gel results in restoring the expression of transcription factor Sox 9 and chondrocyte differentiation markers such as collagen type II and aggrecan [38]. The current study indicated that treatment of cultured chondrocytes with 1, 25 (OH)_2_D_3_ results in restoring of the transcription factor Sox 9, which could indicate chondrocyte re-differentiation and restoration of the chondrocyte phenotype.

The physiological activities of Sox 9 protein were demonstrated by mouse genetic studies which indicated the importance of Sox 9 expression level in determining chondrocyte phenotype during development [39,40]. These studies demonstrated that chondrocyte differentiation and cartilage development were severely affected by knock-out or knock-in of a single allele of Sox 9. Moreover, the campomelic dysplasia is a genetic disorder characterized by multiple developmental abnormalities including cartilage induced by haplo-insufficiency of Sox 9 [18].

In human normal articular chondrocytes, Sox 9 expression was progressively reduced by passage in monolayer cell culture [41]. Moreover its expression is reduced in osteoarthritic chondrocytes compared to the normal articular chondrocytes [23] and has therefore been suggested to contribute in osteoarthritis disease processes by altering the ECM gene expression [42]. Thus, improving the expression of Sox 9 by 1, 25 (OH)_2_D_3_ treatment could provide a new insight into the prevention and/or treatment of osteoarthritis.

In fetal cartilage as well as in mature cartilage, the main role of Sox 9 is to maintain the chondrocyte phenotype in addition to inhibition of hypertrophic chondrocyte differentiation [43]. Therefore, down regulation of Sox 9 expression was suggested to be a precondition for hypertrophic alteration occurring in degenerative cartilage [42,44]. As Sox 9 protein is augmented by 1, 25 (OH)_2_D_3_ treatment, this study suggests that 1, 25 (OH)_2_D_3_ treatment could be utilized to improve the ECM proteins biosynthesis by enhancing the anabolic activities of articular chondrocytes. After further passages, chondrocytes have been shown to exhibit a more pronounced de-differentiated phenotype and lower levels of Sox 9 [41]. Further studies on the effects of 1, 25 (OH)_2_D_3_ upon these cells would be of great interest.

**Figure 2:**
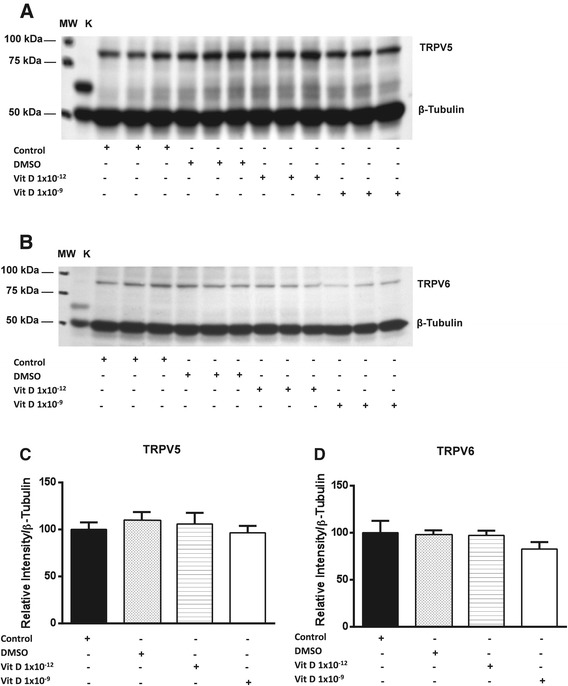
**Expression of TRPV5 and TRPV6 channels in equine articular chondrocytes at the protein level.** The changes in expression of TRPV5 **(A)** and TRPV6 **(B)** channels following treatment with 1, 25 vit D were assessed by densitometry analysis of western blots and normalized to the expression of housekeeping protein (β-Tubulin). Equine kidney (K) lysate was used as a positive control.Levels of expression at the protein level of the TRPV5 **(C)** and TRPV6 **(D)** channels following 24 h of incubation with different doses of 1, 25 (OH)_2_D_3_ in addition to the solvent (DMSO). Data presented as a mean ± S.E.

**Figure 3:**
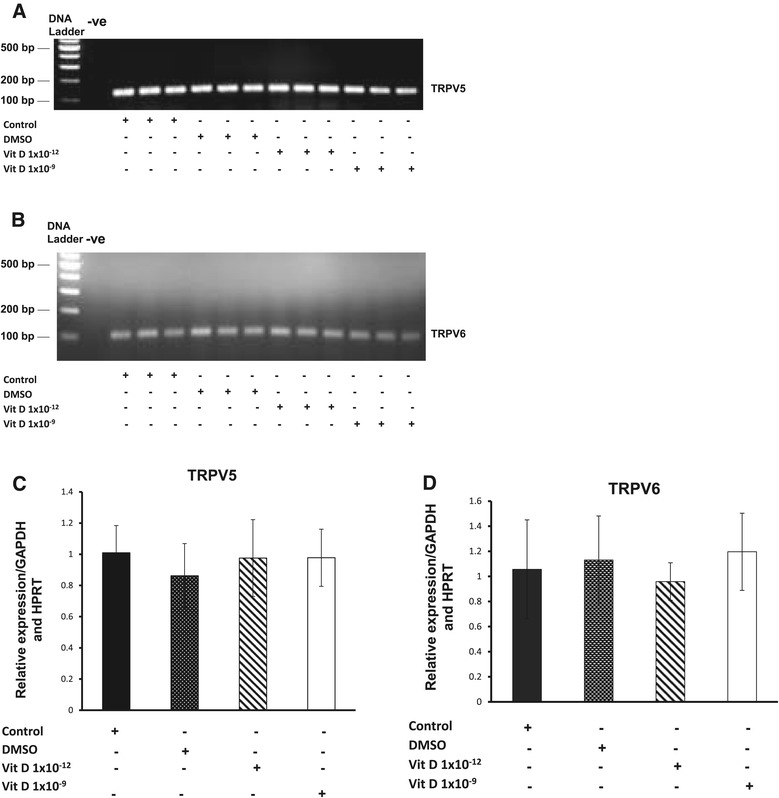
**Expression of TRPV5/6 at the mRNA level.** End point PCR analysis of TRPV5 (A) and TRPV6 (B) ion channel genes on mRNA isolated from equine articular chondrocytes. The graphs show real-time PCR analysis of TRPV5 **(C)** and TRPV6 **(D)** ion channel gene expression levels. Experiments were carried out on equine articular chondrocytes (control) and treated with different doses of 1, 25 (OH)_2_D_3_. GAPDH and HPRT were included as internal controls. Data presented as a mean ± S.E.

We have previously shown that the expresson of both TRPV5 and 6 channels, at the protein level, are inversely related to chondrocyte de-differentiation [24]. In this study we therefore examined the influence of 1, 25 (OH)_2_D_3_ treatment on the expression of TRPV5/6 channels on primary equine articular chondrocytes isolated at passage two. The findings of the current study indicated that no statistically significant changes were observed on the expression of TRPV5 and TRPV6 channels at the protein level following treatment with different doses of 1, 25 (OH)_2_D_3_ (Figure 2). To ensure that 1, 25 (OH)_2_D_3_ had no effect on the expression of these channels we also examined its effect upon their transcript levels but again no significant changes were observed (Figure 3). Previous studies conducted on mouse investigated the effect of 1, 25 (OH)_2_D_3_ on epithelial Ca^2+^ transport including TRPV5 and 6 channels following 1, 25 (OH)_2_D_3_ treatment [45,46]. These studies demonstrated that the expression of TRPV5 and TRPV6 channels in kidney and duodenum were stimulated by binding of VDR (vitamin D receptor) at transcriptional level. TRPV6 was reported to have several classes of VDREs (vitamin D response elements) in humans and the mouse [45,47,48].

In the vitamin D receptor knockout mice model, the level of gene expression pattern of the TRPV6 and 5 channels in duodenum is dramatically down-regulated, in contrast no changes are observed in gene expression of other Ca^2+^ transporters [49,50]. The role of vitamin D in the stimulation of Ca^2+^ transporters has emerged recently. Therefore, the correlation between 1, 25 (OH)_2_D_3_ treatment and Ca^2+^ transport proteins were investigated in several cell types. 1, 25 (OH)_2_D_3_ was demonstrated to increase the expression of TRPV6 channel, calbinding-D9k and PMCA1b genes in Caco-2 cell lines [36,51]. This finding was not consistent with the current study, where no changes were observed on the expression of either TRPV5 or 6 channels at mRNA level or protein levels. As stated above we have previously observed an inverse correlation between TRPV 5 and 6 levels and differentiation state. The current study suggests that the changes in levels may not be directly linked to the de-differentiation process. This however, needs further study as does the mechanism by which Sox-9 is regulated by 1, 25 (OH)_2_D_3_. Ca^2+^ ions and Ca^2+^ channels have been implicated in chondrocyte metabolism [52,53]. In vitro studies show that augmented extracellular Ca^2+^ promotes differentiation toward a hypertrophic phenotype, in contrast Ca^2+^ reduction improves ECM protein synthesis including collagen type II and aggrecan and delays hypertrophy [54]. Therefore, investigating the effect of the 1, 25 (OH)_2_D_3_ on the expression of Ca^2+^ channels could be considered as a candidate for treatment of joint diseases.

The results of the current study do not, however, suggest an involvement of TRPV5/6 ion channels in the regulation of vitamin D mediated up-regulation of sox-9.

## Conclusions

This study examined the effects of the active form of vitamin D (1, 25 (OH)_2_D_3_) upon the expression of transcription factor Sox 9 and the calcium sensitive TRPV 5 and 6 channels, in equine articular chondrocytes, *in vitro*. An increased expression of Sox 9, in these chondrocytes, in response to the active form of vitamin D suggests that this compound acts to inhibit the progressive de-differentiation that is normally observed in these cells. It is also supportive of previous studies indicating that 1α, 25-dihydroxyvitamin D_3_ can have a protective effect upon cartilage in animals *in vivo*. There was no effect of vitamin D (1, 25 (OH)_2_D_3_) upon the expression of either TRPV channel at either the protein or mRNA level. We have previously observed a correlation between the degree of differentiation and the expression levels of TRPV 5/6 and suggested that these ion channels may have a direct interaction with the differentiation process *in vitro*. The data in the present study do not support this.
